# Under expression of the Sonic Hedgehog receptor, Patched1 (PTCH1), is associated with an increased risk of local recurrence in squamous cell carcinoma of the vulva arising on a background of Lichen Sclerosus

**DOI:** 10.1371/journal.pone.0206553

**Published:** 2018-10-31

**Authors:** Jason Yap, Richard Fox, Natalie Narsia, Sonia Pinheiro-Maia, Rachel Pounds, Ciaran Woodman, David Luesley, Raji Ganesan, Sean Kehoe, Christopher Dawson

**Affiliations:** 1 Birmingham Cancer Research UK Cancer Centre, Institute of Cancer and Genomic Sciences, University of Birmingham, Birmingham, West Midlands, United Kingdom; 2 Pan Birmingham Gynaecological Cancer Centre, City Hospital, Birmingham, West Midlands, United Kingdom; 3 Department of Clinical and Molecular Pathology & Laboratory of Molecular Pathology, Palacky University, Olomouc, Moravia, Czech Republic; 4 Department of Histopathology, Birmingham Women’s NHS Foundation Trust, Birmingham, West Midlands, United Kingdom; Rudjer Boskovic Institute, CROATIA

## Abstract

**Objective:**

Dysregulation of the Hedgehog (Hh) pathway has been described in a variety of cancers, including cervical cancer, a disease which shares a common aetiology with vulval squamous cell carcinoma (VSCC). Here, we investigate a large number of primary VSCC cases for evidence of Hedgehog pathway activation and examine the implications of pathway activity on clinical outcomes in a cohort of patients with primary VSCC.

**Methods:**

Archival histology blocks containing VSCC and histologically normal adjacent epithelium were retrieved from a cohort of 91 patients who underwent treatment for primary VSCC. Immunohistochemistry staining was undertaken to assess for the expression of key Hh pathway components (SHH, PTCH1, GLI1). A competing risks statistical model was used to evaluate the implications of the levels of key Hh pathway components on clinical outcomes.

**Results:**

We show that 92% of primary VSCC cases over-expressed one or more components of the Hh signalling pathway when compared to the adjacent normal epithelium. While expression of SHH and GLI1 did not correlate with any clinicopathological criteria, over- or under-expression of PTCH1 was associated with a reduced or increased risk of developing a local disease recurrence, respectively. In VSCC arising on a background of Lichen Sclerosus, the risk of local recurrence was potentiated in cases where PTCH1 was under-expressed.

**Conclusions:**

Our findings reveal, for the first time, that the Hh pathway is activated in VSCC and that PTCH1 expression can be used as a biomarker to stratify patients and inform clinicians of the risk of their local recurrence, particularly in cases of VSCC associated with LS.

## Introduction

Vulval cancer comprises 6% of all gynaecological malignancies in the UK, with squamous cell carcinoma (VSCC) making up 90% of all cases [1]. It is predominantly a disease of the elderly, with three-quarters of cases affecting those aged over 60 years, making radical treatment challenging due to specific age-related comorbidities. Surgery remains the most effective treatment modality for VSCC, and the current surgical paradigm aims to excise at least 1.5cm of tumour-free skin along with the primary tumour to avoid local recurrence [2]. However, recently published studies, including ours, have shown that inadequate surgical margins are not associated with the development of local vulval recurrence (LVR) as long as the tumour is entirely excised [3,4]. In our previous study, we found that most local recurrences occurred in cases where optimal surgical margins had been achieved. Furthermore, we also demonstrated that VSCC arising in a background of Lichen Sclerosus (LS), a chronic inflammatory dermatosis affecting the whole vulva, were more likely to develop an LVR after primary treatment [5]. Our findings, together with others, suggest that these “recurrent tumours” most likely constitute a new primary tumour that arises in a field of a molecularly altered epithelium. While the molecular pathways linked to disease recurrence are yet to be fully defined, the Hedgehog (Hh) pathway is of particular interest in this context, given that Hh pathway dysregulation has been described in cancers associated with highrisk human papillomavirus (HR-HPV) and chronic inflammation [6,7]; both of which are recognised independent aetiological factors for VSCC [4].

In the canonical Hh signalling cascade, binding of Sonic Hedgehog (SHH) to the Hedgehog receptor, PTCH1, relieves its repression on Smoothened (SMO), a G-protein coupled receptor. This results in stabilisation and nuclear translocation of the GLI proteins and pathway activation [8]. In adulthood, this cell signalling pathway is usually repressed, but its activity is maintained in certain stem cell populations to promote tissue renewal and regeneration. Dysregulation of the Hh pathway has been described in a variety of cancers from multiple tissue types [9]. In gynaecological cancers, as with other malignancies, aberrant Hh pathway activation is associated with poor treatment outcomes or the development of chemoresistance [10,11,12]. To this end, we have undertaken a study to investigate the status of Hh pathway activity in VSCC using our previously published cohort of patients diagnosed with primary VSCC [5] and examined the possible implications of Hh pathway activation to clinicopathological criteria.

## Materials and methods

Study population: This included 91 primary cases of VSCC diagnosed between 2000 and 2008 and managed in the Pan Birmingham Gynaecological Cancer Centre. Comprehensive information of the cohort’s demography, behaviour, clinicopathological variables, HPV genotyping and treatment outcomes are already published [5]. Time to recurrence/death was measured from the date of primary treatment to the date of clinical follow up where the diagnosis was made based on either histological confirmation of invasive disease, clinically diagnosed recurrence, clinically unambiguous evidence of disease progression, or death. All patients were followed up continuously for 56 months. As outlined in our previous study and having previously observed 2 different patterns of local recurrence, we have dichotomised LVR into local relapse (LR), a tumour which recurs within 2 cm of the primary tumour, and second field tumour (SFT), a tumour which recurs >2 cm away from the primary tumour [5].

Immunohistochemical (IHC) staining: Archival formalin-fixed paraffin embedded (FFPE) histology blocks consisting of the primary tumour and their corresponding histologically adjacent normal vulval epithelium were retrieved and 4-micron sections processed for immunohistochemical staining as previously described [13]. The antibodies used to stain for Hh pathway components included antibodies specific for SHH, PTCH1 and GLI1 are listed in S1 Table. The expression of Hh pathway components was quantified using the H-score system [13,14]; and over- or under-expression of Hh pathway components in the tumour were defined by a H-score of ≥1 when compared to the respective adjacent normal epithelium. Histology and staining were comprehensively reviewed by a gynaecological cancer specialist pathologist, RG.

HPV-genotyping: This was performed on formalin fixed paraffin embedded blocks of VSCC using standard PCR methods as previously described to detect the presence of HPV16 and 18 strains [15].

Statistical analyses: Expression of Hh pathway components in tumour were compared to that of the corresponding measurements in adjacent normal epithelium using the Wilcoxon signed rank test for paired observations. The tumour expression of GLI1 and PTCH1 were also correlated with SHH using linear regression. Survival time was defined as time to recurrence or death, with death from primary disease contributing as a recurrence, or date last known to be alive for surviving patients. Death precludes the observation of disease recurrence, and hence standard survival methodology, Kaplan Meier estimates alongside Cox proportional hazards modelling, yield biased risk estimators. As such, competing risk models were derived to take into consideration that most women in our cohort were elderly and likely to die of causes unrelated to VSCC [16]. We quantify the impact of patient, disease and treatment characteristics upon incidence of recurrent disease, and quantify incidence modification using a Sub-Hazards-Ratio (SHR) with 95% confidence interval (95% CI). Exploratory univariable models were constructed, followed by multivariable models with prognosticators selected based on Akaike information criterion (AIC). For the univariable analyses, we considered predictors that were significant at the p = 0.1 to be potential incidence modifiers. The assumptions associated with such models were tested, and time-dependent effects were explored upon indication non-proportional sub-hazards. The strict interpretation of a sub-hazard ratio is discussed later. For simplicity, we interpret a significant SHR as an association with incidence modification. Those factors considered were: Hh pathway components (SHH, PTCH1, GLI1); vulvar intraepithelial neoplasia (VIN); LS; age; smoking status; disease stage; disease focality; groin node metastasis; groin node surgery; lymphovascular space invasion (LVSI); excision margins; histology grade; chemo/radiotherapy; radiotherapy for sub-optimal surgery; type of surgery. Further univariable analysis was also undertaken to assess cytosolic GLI1 or combined cytosolic and nuclear GLI1 expression in tumour and adjacent normal tissue with respect to the incidence of recurrent disease. Statistical analyses were performed using Stata V14.

Details of Ethics Approval: This study was approved by the National Research Ethics Service Committee West Midlands–Solihull (Reference 11/WM/0070). All samples were anonymised.

## Results

### Study population

A summary of the distribution of the clinicopathological variables for all the 91 patients is shown in Table 1. The mean age of the women in this cohort was 74 years (IQR 63–81). As this is a retrospective cohort, all patients had surgico-pathological staging according to the FIGO 1998 staging criteria. The staging system was simplified into early (stage 1 and 2) and advanced stage (stage 3 and 4) to facilitate statistical analysis: 55 (60%) patients presented with early stage disease and 36 (40%) had advanced stage disease. In 40 (44%) patients LS was found adjacent to their main VSCC and VIN was found adjacent to the main tumour in 66 (72.5%) patients. HPV genotyping revealed that 52 (57%) tumours tested positive for HPV16/18 subtypes with the remaining 39 (43%) of the cohort testing negative.

**Table 1:** Distribution of clinicopathological variables in the Birmingham VSCC cohort.

Variable		Number of cases n (%)
Age (years)	Mean	74.0 (IQR 63.0, 81.0)
Smoking status	Smoker/Ex-Smoker	26 (28.6)
	Never smoker	48 (52.7)
	Missing	17 (18.7)
Stage (simplified)	1/2 (Early)	55 (60.4)
	3/4 (Advanced)	36 (39.6)
Tumour Size	< 2cm	9 (9.9)
	2-<4cm	37 (40.7)
	4<6cm	24 (26.4)
	> = 6cm	13 (14.3)
	Missing	8 (8.8)
Focality	Unifocal	73 (80.2)
	Multifocal	18 (19.8)
Histology grade	Well	17 (18.7)
	Moderate	31 (34.1)
	Poorly	37 (40.7)
	Not graded	6 (6.6)
LVSI	Yes	38 (41.8)
	No	50 (54.9)
	Missing	3 (3.3)
LS, +/- VIN	LS, +/- VIN	40 (44.0)
	No LS	51 (56.0)
VIN	uVIN, dVIN or ungraded	66 (72.5)
	No VIN	25 (27.5)
HPV 16/18 E6	Positive	52 (57.1)
	Negative	39 (42.9)
Groin node metastasis	Yes	26 (28.6)
	No	65 (71.4)
Excision Margins	Optimum	48 (52.7)
	Sub-optimum	33 (36.3)
	Incomplete	7 (7.7)
	Missing	3 (3.3)
Groin Node Surgery	SNLB/GLND	70 (76.9)
	No nodal surgery	21 (23.1)
Chemo/Radio-therapy	Yes	28 (30.8)
	No	63 (69.2)

Surgical excision was the main modality of treatment with 89 (98%) patients undergoing radical excision to remove their primary tumour, while 70 (77%) patients had groin lymphadenectomies or sentinel lymph node biopsies. Of the 89 patients who had surgery, 48 (54%) had “optimal tumour resection” with tumour free margins of at least 8mm or more; 33 (37%) had tumour free margins of less than 8mm; tumours were incompletely excised in 7 (8%) cases; and resection margins were unavailable for 1 patient. Primary/neo-adjuvant/adjuvant radiotherapy with or without chemotherapy was administered in 28 patients (31%).

### Hedgehog pathway components (SHH, PTCH1 and GLI1) are over-expressed in VSCC

**Fig 1:**
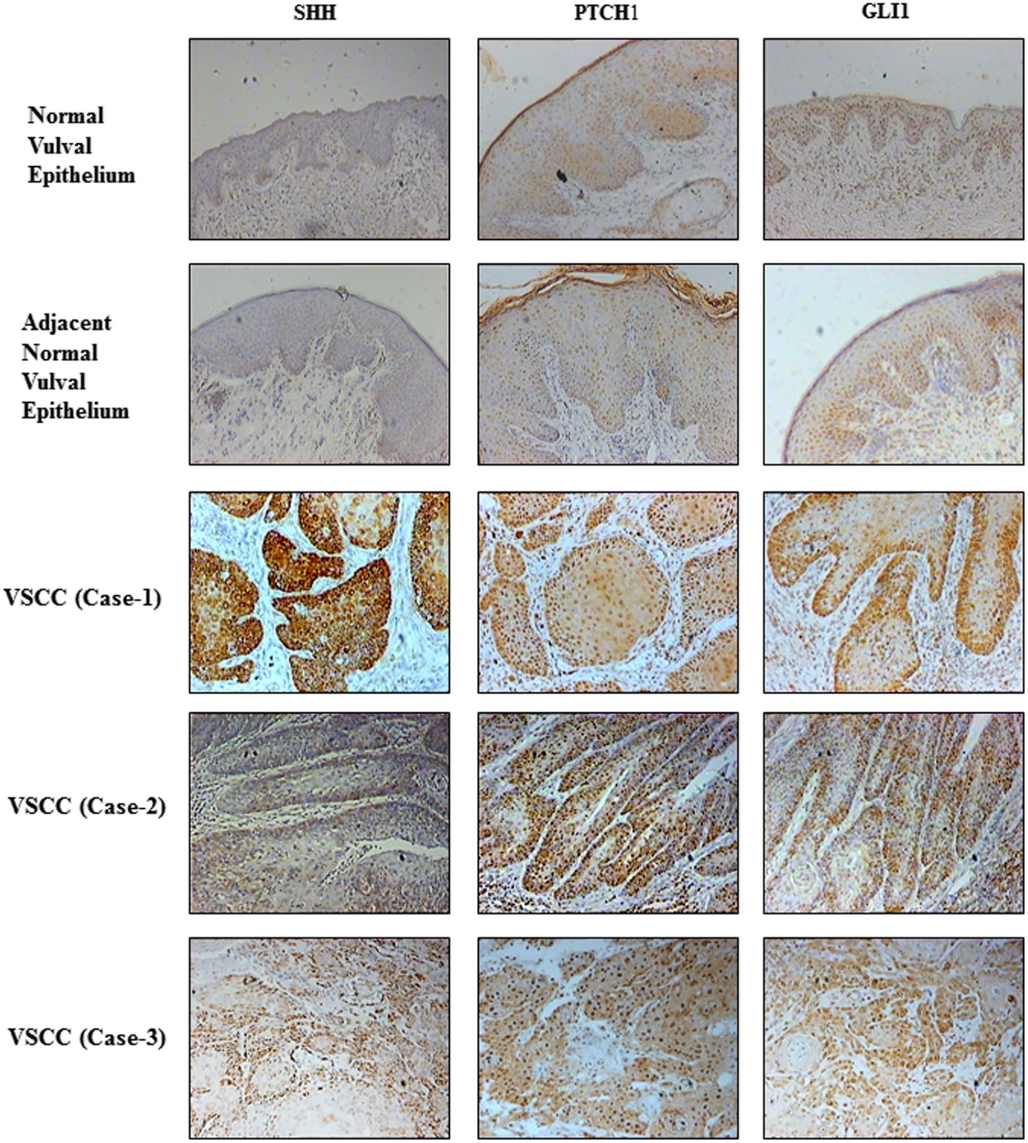
The Hedgehog pathway is aberrantly activated in VSCC compared to normal vulval epithelium. Representative immunohistochemical staining showing differential expression of SHH ligand, PTCH1, and GLI1, in normal vulval squamous epithelium, normal tumour-adjacent vulval epithelium (upper panels) and three primary VSCC cases (lower panels) (original magnification x200). Insets show higher magnification of the same sections (magnification x400).

IHC staining was undertaken to evaluate the expression of Hh pathway components (SHH, PTCH1 and GLI1) in the primary tumour and their respective adjacent histologically normal epithelium. In all cases, expression of SHH ligand was localised to the cytosol; PTCH1 to the membrane, cytosol and nucleus; and GLI1 to the cytosol and nucleus (Fig 1 and S1 Fig for high resolution images).

**Fig 2:**
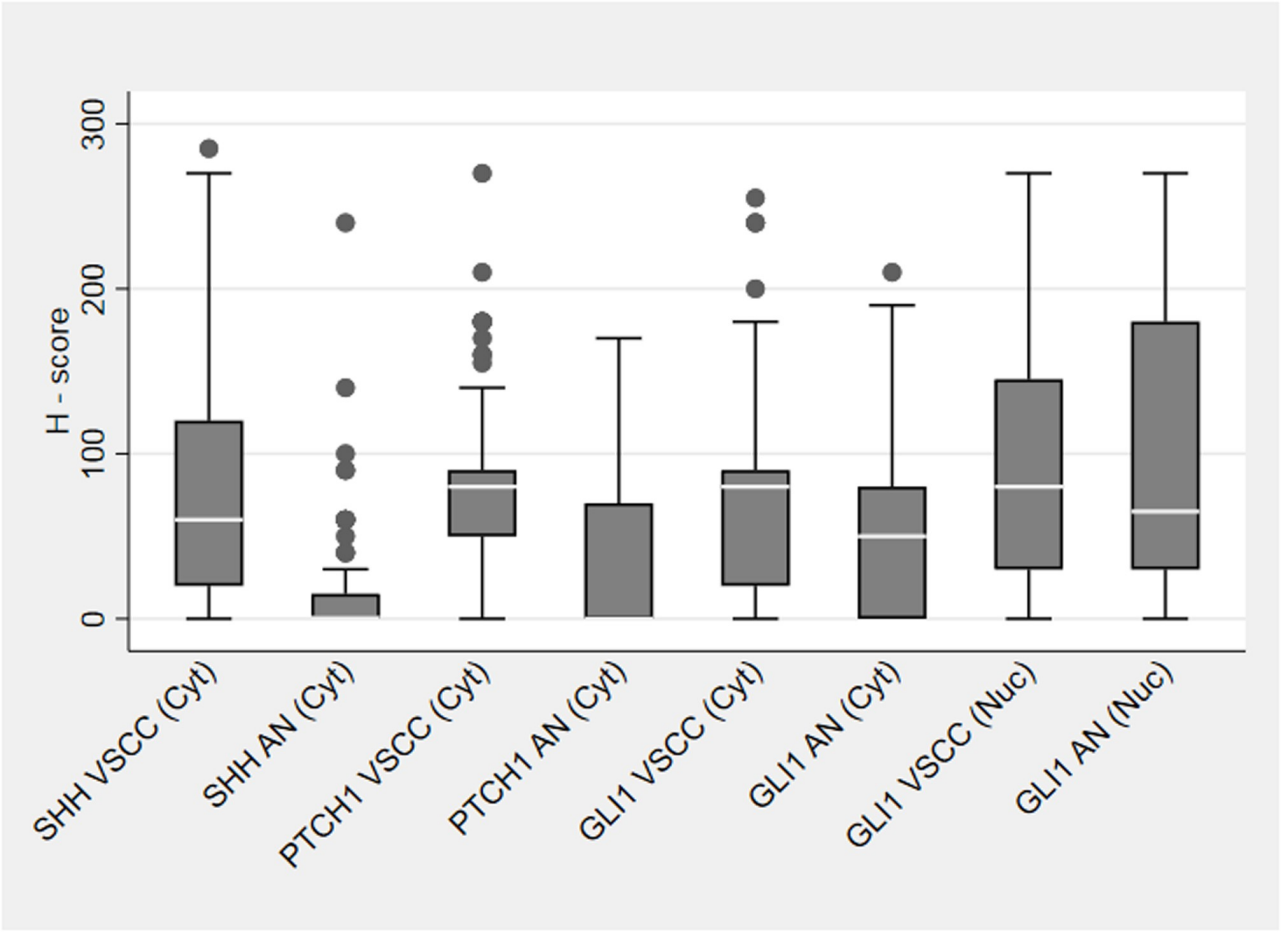
Box & Whiskers plot showing the expression of SHH, PTCH1 and GLI1 in the main VSCC tumour and respective adjacent normal epithelium. AN = adjacent normal epithelium, Cyt = cytosolic, Nuc = nuclear.

Compared to adjacent histologically normal epithelium, 84 (91%) VSCC cases exhibited over-expression of at least one Hh pathway component (SHH or GLI1 or PTCH1): SHH ligand was over-expressed in the primary tumour in 73 (80%) cases; 47 (52%) cases showed a cytosolic over-expression of GLI1, and PTCH1 was over-expressed in 53 (58%) cases. Paired non-parametric comparisons of average tumour expression showed an increase in the expression of SHH ligand (p<0.001), PTCH1 (p<0.001) and cytosolic GLI1 (p = 0.002) in the primary tumour compared to the adjacent histological normal epithelium (Fig 2). Although the average levels of nuclear GLI1 expression were higher in VSCC compared to adjacent normal epithelial, the difference was not statistically significant (p = 0.982). There was a weak positive correlation between the levels of SHH and its downstream targets: PTCH1 and GLI1 (subdivided into cytosolic GLI1 and nuclear GLI1), with R-squared values of 9%, 11% and 4% respectively. The R-squared value for the correlation between PTCH1 and GLI1 was 26%.

### Increased expression of PTCH1 in VSCC is associated with a reduced risk of developing a local disease recurrence

The median follow-up for patients who were still alive was 8.5 years, and 35 (38%) patients within this cohort were still alive at the time this study was undertaken. Of these patients, 26 (29%) women had a total of 44 episodes of LVR: 17 (19%) had LR, and 18 (20%) had SFT. Disease-specific death (DSS) was reported in 28 (31%) women. Seven women who had residual disease following primary intervention were omitted from the analyses of recurrence outcomes.

The univariable analyses (Table 2) indicated that Hh pathway component expression and clinical determinants which predispose to LVR were the presence of LS adjacent to VSCC (SHR = 3.96 95% CI 1.55, 10.10; p = 0.004) and testing negative for HR-HPV (SHR 2.60 95% CI 1.14, 5.92; p = 0.022). For LR, the sole determinant was the presence of adjacent LS (SHR = 5.35 95% CI 1.50, 19.00; p = 0.010), while over-expression of PTCH1 was found to protect against LR (SHR 0.25 CI 0.080, 0.778, p = 0.0017). For DSS, the clinical determinants associated with adverse outcomes were advanced disease stage (SHR 2.58 95% CI 1.24, 5.33; p = 0.011) and groin node metastasis (SHR 2.96 95% CI 1.42, 6.18; p = 0.004).

**Table 2:** Univariable analyses.

	Local Vulva Recurrence (LVR)		Local Relapse (LR)		Second Field Tumour (SFT)		Disease Specific Survival (DSS)	
Covariate	HR (95% CI)	P-value	HR (95% CI)	P-value	HR (95% CI)	P-value	HR (95% CI)	P-value
SHH: Ref Cat = No over-expression								
Over-expressed	**6.62 (0.96, 45.80)**	**0.056**	3.86 (0.54, 27.86)	0.180	2.07 (0.52, 8.19)	0.302	1.59 (0.57, 4.39)	0.372
PTCH1: Ref Cat = No over-expression								
Under-expressed	0.85 (0.34, 2.13)	0.728	**0.25 (0.08, 0.78)**	**0.017**	1.16 (0.35, 3.78)	0.810	0.73 (0.32, 1.65)	0.445
Unavailable	1.18 (0.31, 4.43)	0.811	0.80 (0.18, 3.64)	0.772	0.70 (0.08, 6.20)	0.748	1.07 (0.39, 2.99)	0.892
GLI1(Nuc): Ref Cat = No over-expression								
Over-expressed	1.20 (0.51, 2.81)	0.675	1.37 (0.46, 4.10)	0.577	0.72 (0.26, 2.04)	0.540	1.12 (0.51, 2.47)	0.772
Unavailable	0.99 (0.22, 4.42)	0.988	0.86 (0.10, 7.41)	0.890	Complete	Complete	0.72 (0.16, 3.15)	0.662
LS, +/- Vin: Ref Cat = No LS								
LS, +/- Vin	**3.96 (1.55, 10.10)**	**0.004**	**5.35 (1.50, 19.00)**	**0.010**	2.44 (0.90, 6.59)	0.078	1.56 (0.75, 3.25)	0.231
Age (years)	1.01 (0.99, 1.03)	0.281	1.01 (0.98, 1.03)	0.617	1.01 (0.99, 1.04)	0.316	1.02 (0.99, 1.04)	0.163
Smoking status: Ref Cat = No								
Smoker/Ex-Smoker	0.73 (0.29, 1.84)	0.502	1.24 (0.41, 3.73)	0.702	0.51 (0.15, 1.81)	0.300	0.71 (0.28, 1.80)	0.465
Unavailable	0.58 (0.16, 2.11)	0.407	0.74 (0.15, 3.62)	0.714	0.27 (0.03, 2.21)	0.221	1.20 (0.47, 3.09)	0.707
Stage (simplified): Ref Cat = 1/2								
3/4	1.04 (0.45, 2.38)	0.930	0.80 (0.28, 2.32)	0.679	0.88 (0.29, 2.61)	0.813	**2.58 (1.24, 5.33)**	**0.011**
Disease Multifocal: Ref Cat = No								
Yes	1.46 (0.57, 3.70)	0.431	1.54 (0.49, 4.86)	0.461	0.65 (0.14, 2.94)	0.578	0.57 (0.21, 1.59)	0.284
Groin node involvement: Ref Cat = No								
Yes	1.50 (0.64, 3.50)	0.347	1.36 (0.47, 3.95)	0.567	1.04 (0.33, 3.29)	0.947	**2.96 (1.42, 6.18)**	**0.004**
LVSI: Ref Cat = No								
Yes	0.48 (0.19, 1.20)	0.118	0.39 (0.13, 1.21)	0.103	0.93 (0.32, 2.68)	0.895	0.66 (0.31, 1.40)	0.279
HPV 16/18: Ref Cat = Positive								
Negative	**2.60 (1.14, 5.92)**	**0.022**	2.20 (0.79, 6.13)	0.132	1.98 (0.70, 5.62)	0.201	1.49 (0.72, 3.09)	0.284
VIN: Ref Cat = Any VIN (u.d, ungraded)								
No VIN	1.15 (0.49, 2.71)	0.743	1.31 (0.45, 3.75)	0.620	0.71 (0.20, 2.53)	0.601	2.01 (0.95, 4.28)	0.068
Excision Margins: Ref Cat = Incomplete								
Optimum	1.03 (0.24, 4.35)	0.966	0.56 (0.12, 2.63)	0.460	Unable to compute as 0 cases in “Incomplete” group		0.51 (0.12, 2.10)	0.349
Sub-optimum	0.86 (0.19, 3.82)	0.839	0.50 (0.10, 2.52)	0.398			0.82 (0.20, 3.32)	0.781
Unavailable	Complete		Complete				7.18 (0.62, 83.16)	0.115
Histology grade: Ref Cat = Well								
Moderate	0.51 (0.14, 1.82)	0.301	0.55 (0.14, 2.18)	0.396	0.38 (0.08, 1.77)	0.216	1.12 (0.35, 3.60)	0.844
Poorly	0.86 (0.30, 2.49)	0.782	0.46 (0.12, 1.79)	0.260	0.63 (0.18, 2.20)	0.468	2.15 (0.77, 6.04)	0.145
Not graded	1.61 (0.44, 5.94)	0.474	2.61 (0.63, 10.79)	0.187	0.63 (0.08, 5.18)	0.669	1.23 (0.26, 5.78)	0.791
Tumour Size: Ref Cat = < 2cm								
2-<4cm	0.81 (0.22, 3.02)	0.756	1.05 (0.22, 4.95)	0.952	0.51 (0.13, 1.97)	0.332	0.71 (0.21, 2.36)	0.578
4-<6cm	0.62 (0.15, 2.55)	0.510	0.32 (0.05, 2.24)	0.249	0.33 (0.07, 1.63)	0.174	1.68 (0.52, 5.41)	0.388
≥6cm	0.39 (0.06, 2.62)	0.334	0.32 (0.03, 3.77)	0.364	0.20 (0.02, 2.06)	0.177	1.43 (0.33, 6.14)	0.628

Multivariable analyses, with adjustment for confounding, revealed that under-expression of PTCH1 in VSCC was associated with an increased incidence of LR (SHR = 4.00 95% CI 1.29, 12.50; p = 0.017) and vice versa. As reported in our previous analysis [5], the presence of LS adjacent to the primary VSCC remained an independent clinical determinant associated with the development of LR. For DSS, multivariable analyses revealed no confounding, and hence groin node metastasis remained the only clinical determinant that predicts survival, with those patients with positive groin nodes having a poorer outcome, again, a result similar to our previous analysis.

There was no correlation between GLI1 over-expression and LVR, LR, SFT or DDS. In addition, further analysis did not reveal any association between Hh pathway component overexpression with HR-HPV status. As reported in our previous study, HR-HPV status neither influenced disease recurrence nor survival.

### PTCH1 under expression further potentiates the risk of LR in VSCC arising in the background of LS

**Fig 3:**
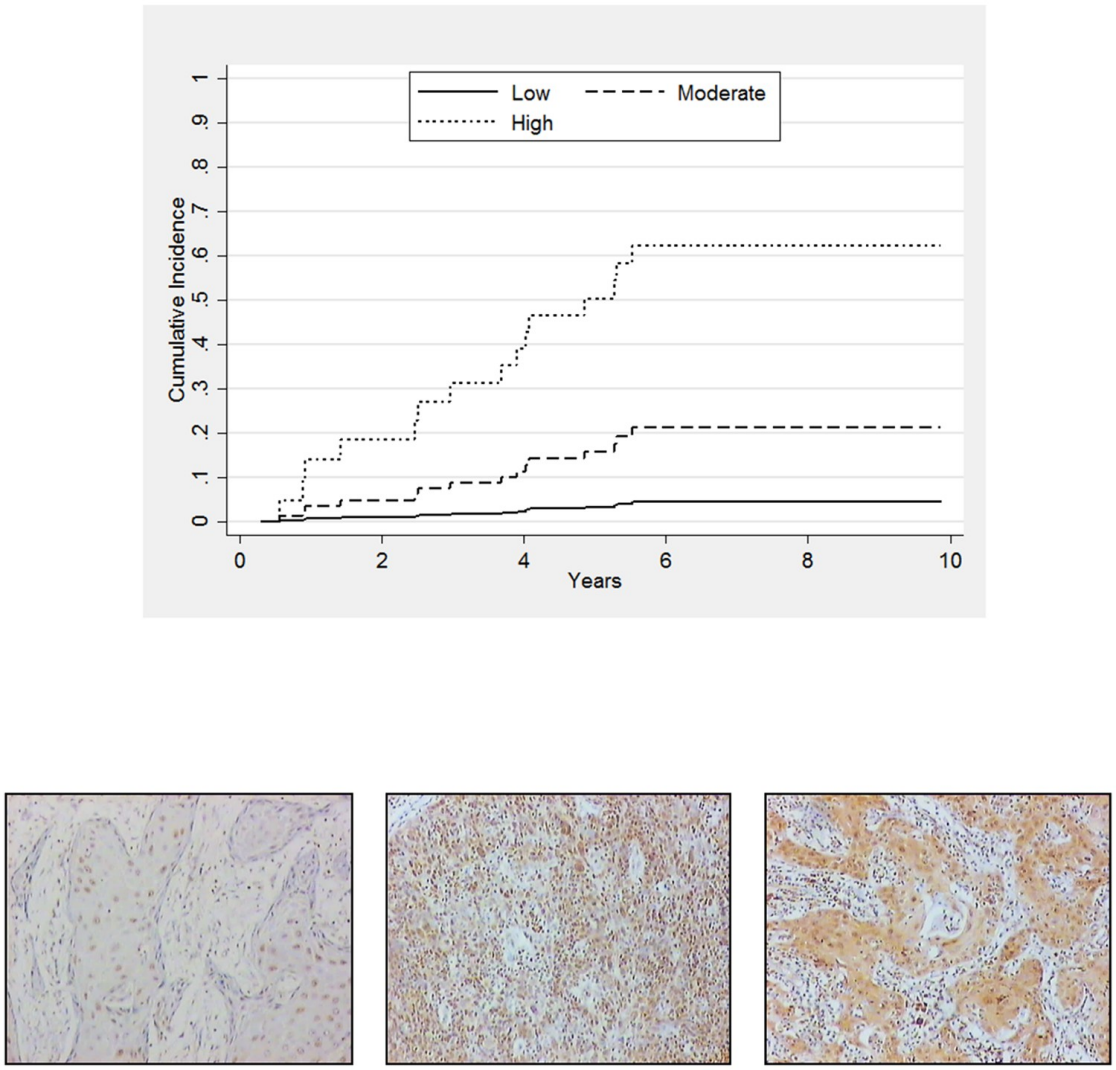
(A) Cumulative incidence plots showing incidence associated with PTCH1 expression and/or the presence of adjacent LS identified through multivariable analyses for time to local relapse (LR). The incidence of LR within 5 years following primary treatment is significantly increased in the high-risk group compared to the moderate- and low-risk groups. High risk: LS & PTCH1 under-expression; Moderate-risk: Either LS or PTCH1 under-expression; Low-risk: Neither LS nor PTCH1 under-expression. (B) Representative IHC staining of VSCC cases displaying (a) low, (b) medium and (c) high levels of PTCH1.

As the presence of adjacent LS and the status of PTCH1 expression are both associated with an increased incidence for LR, we next evaluated the impact of PTCH1 expression in VSCC cases associated with and without LS. We stratified our patients into three risk groups: 1) High-risk: VSCC associated with LS and showing PTCH1 under-expression; 2) Moderate-risk: a tumour either arising from LS or showing PTCH1 under-expression; 3) Low-risk: tumour neither associated with LS nor showing PTCH1 under-expression. By stratifying patients according to the level of PTCH1 expression and association with LS, we demonstrated that following adjustment for patient age, LR is further potentiated in patients in the high-risk group when compared to those in the low-risk group, SHR of 21.03 (95% CI: 2.08, 212.93; p = 0.010), (Fig 3A and 3B). Further model evaluation revealed that the relative difference in sub-hazards between the moderate and low-risk groups was not constant, that is, a statistically significant time-dependency was observed. This indicated that, while the risk of LR between the moderate- and low-risk groups did not differ in the first 12 months following primary surgery, the moderate-risk group are exposed to increased relative incidence as time progresses (S2 Table).

## Discussion

Our study demonstrates for the first time, that key components of the Hh pathway are frequently over-expressed in VSCC compared to the adjacent normal epithelium, implying aberrant activation of the pathway in VSCC. Furthermore, we found that levels of the Hh receptor, PTCH1, could predict the relative risk of developing an LR.

In our previous study, we showed that local recurrence in VSCC is not caused by failure to achieve adequate tumour-free surgical margins and proposed that LVR may be driven by underlying molecular changes in residual epithelium left behind after surgery [4]. Here, we show that the Hh signalling pathway, whose dysregulation has recently been described in cervical cancer [10], is also aberrantly activated in primary VSCC. Although there are a number of ways in which the Hh pathway can become dysregulated in cancer [9], it is unclear at this stage what mechanisms are responsible for Hh pathway activation in VSCC. In situations where pathway dysregulation plays a direct role in epithelial carcinogenesis, over-expression of SHH ligand, mutations in PTCH1 or SMO, can all activate the Hh pathway. In the case of basal cell carcinoma (BCC), Hh dysregulation is driven by a mutations in the PTCH1 gene, which blocks its ability to repress SMO [17]. Although our study did not examine VSCC tumours for evidence of PTCH1 or SMO mutations, we speculate that overexpression of SHH ligand most likely constitutes the mechanism for pathway activation in VSCC, given that in cases where SHH was over-expressed, there was a corresponding upregulation of PTCH1 and GLI1. Similar findings have been reported for cervical cancer, which shares a common aetiology to at least half of the VSCC cases [10].

In many Hh-driven cancers, the degree of pathway activation has been shown to influence disease outcomes, suggesting that key Hh pathway components may serve as clinically useful biomarkers to inform clinicians about treatment responses [18]. Using our previously published patient follow-up data, univariable and multivariable analyses were constructed to interrogate the association of Hh pathway components with various clinicopathological criteria. Of the major criteria investigated, our study revealed that over-expression of PTCH1 was associated with a reduced incidence of LR, while under-expression was associated with an increased incidence. These findings are consistent with PTCH1 functioning as a tumour suppressor gene [18]. PTCH1 functions as a negative regulator of the Hh-signalling pathway by repressing downstream signalling from SMO. As a direct target of the GLI transcription factors, high levels of PTCH1 are linked to elevated Hh pathway activation, where PTCH1 functions in a negative feedback loop to attenuate Hh signalling. Mutations or loss of PTCH1 expression, through epigenetic silencing, leads to constitutive activation of the pathway and expression of Hh target genes [19]. Findings from this study suggest that the loss of PTCH1 expression on a background of chronic Hh pathway activation can promote LVR and LR, implicating a role for Hh signalling in disease recurrence.

Interestingly, we found in our sub-analysis that PTCH1 under-expression further potentiates the risks of LR in VSCC arising in a background of LS, indicating that we can further stratify patients who are at risk of developing an LR, using PTCH1 as a biomarker. Our observation also suggests that Hh pathway dysregulation may be significant in the setting of LS, given that chronic inflammation can aberrantly active the Hh pathway [7]. As only 4–5% of LS progress to VSCC [20], it would be interesting to establish whether Hh pathway dysregulation plays a role is in those cases which progress to cancer. Those patients in the Moderate-risk group were still at risk of developing an LR over time compared to those in the Low risk group, suggesting that either PTCH1 under-expression or LS are independent markers associated with LR. We advocate, therefore, that these patients should be followed up more frequently than patients whose tumours do not show PTCH1 under-expression or do not have underlying LS. This observation fits with our multivariable analysis indicating that both of these are independent risk factors but could potentiate patients’ risk of developing an LR when both are present.

At this stage, it is unclear whether further corruption of the Hh pathway is required for LVR and LR in VSCC. In BCC, a number of Hh pathway-dependent mechanisms have been identified which contribute to tumour recurrence in response to treatment with SMO inhibitors. While mutations in SMO are commonly observed [21, 22], others are associated with copy number changes in SUFU or GLI2 [23]. Recently, a novel mechanism has been identified which involves non-canonical activation of the Hedgehog pathway through a mechanism involving serum-response factor (SRF) and megakaryoblastic leukaemia 1 (MKL1)-mediated activation of GLI1 [24]. While interrogation of such mechanisms is beyond the scope of this study, further studies will investigate whether these or similar mutations or pathway corruption are involved in VSCC recurrence. A major strength of our study is that we compared the levels of individual Hh pathway components (SHH, PTCH1, and GLI1) in primary tumours with tumour adjacent normal epithelium. Such an analysis provides a valuable internal control for each case, thereby improving the accuracy of our study. Although our study relates to a retrospective cohort with a monocentric design, much like other studies performed in rare diseases, we believe that our cohort has a comprehensive clinicopathological and follow up data that allow us to interrogate the relevance of Hh pathway expression in VSCC robustly.

Despite the clear-cut demonstration that key components of the Hh pathway were over-expressed in VSCC, the question remains as to how Hh pathway activation contributes to disease pathogenesis. This is pertinent given that expression of GLI1 did not correlate with any specific disease parameter. In this respect, our findings are at odds with recent studies performed on cervical cancer, where over-expression of key Hh components, in particular, GLI1, correlated with disease stage, tumour grade and lymph node involvement [10, 25, 26]. Given the complex pathobiology of VSCC, with the disease arising from HPV-dependent and independent routes, further studies are required to unravel the underlying mechanism(s) which underpin the aberrant expression of the Hedgehog pathway in VSCC, and whether pathway inhibition offers any therapeutic benefit. As in our previous study, we found no compelling evidence to suggest that HR-HPV status influences disease outcome in VSCC [5]. The link between Hh pathway activation and HR-HPV remains to be elucidated. Although we found no association between HR-HPV status and Hh pathway component expression, our study did not interrogate the transcriptional status of the virus, which we believe is beyond the scope of this study. An additional limitation of our study was our inability to stratify the non-neoplastic epithelial disorders (LS, uVIN, dVIN) found adjacent to VSCC into specific groups, given that LS and VIN were frequently found to co-exist in the same tissue specimens. As the numbers in each sub-group were too small to provide a comparison for meaningful statistical analysis, we have grouped all patients with LS (irrespective of the presence of uVIN/dVIN) and compared these against the two other groups, as described in our previous study [5].

We advocate the use of competing risk analyses because patients with VSCC were elderly and likely to die of causes unrelated to their cancer; therefore, an unrelated death is a competing event. We observed in our sub-analysis, comparing the risk of LR in patients stratified into high-, moderate- and low-risk based on PTCH1 expression and the presence of adjacent LS, that women in the low-risk group were more likely to die of causes other than VSCC. Nevertheless, taking into account our competing risk model, we do not believe that this is likely to influence our overall analysis.

## Conclusion

Our study shows, for the first time, that the Hh signalling pathway is aberrantly activated in a significant proportion of VSCC, irrespective of their high-risk HPV status. We show that PTCH1 may serve as a useful biomarker to stratify patients into different risk groups, especially those with LS, so that patient management and follow-up can be tailored accordingly to their relative risk. Ultimately, a multicentre prospective study is required to validate the usefulness of PTCH1 as a biomarker for risk stratification.

Furthermore, our findings offer an opportunity for us to explore and develop novel therapies which target the Hh pathway, where the effectiveness of current chemotherapy for the treatment of VSCC remains questionable and is not tolerated by most patients. In recent years, a number of Hh pathway inhibitors have been evaluated in clinical studies. One such drug, Vismodegib, is licensed by the FDA for the treatment of advanced or metastatic cases of BCC, cancer driven by PTCH1 mutations [27]. Future *in vitro* studies will determine whether Hh-positive VSCC-derived cell lines respond to Hh inhibitors and whether they can be used in the neoadjuvant or adjuvant setting alongside conventional chemotherapy.

## Supporting information

S1 Fig Higher power images from immunohistochemical staining (in Fig 1) showing stronger expression of cytosolic SHH ligand, and both cytosolic and nuclear staining of PTCH1 and GLI1, in cells from a primary VSCC (lower panels) compared to normal vulval squamous epithelium (upper panels) (original magnification x400). (TIF)

S1 Table List of antibodies used for immunohistochemical staining. (TIF)

S2 Table Competing risks models for LR adjusted for (mean centred) patient age, and detailing the time-dependency of the sub-hazard in the moderate-risks group. (TIF)

## References

[1] Cancer Research UK (CRUK). Vulval Cancer Incidence Statistics. 2014. http://www.cancerresearchuk.org/health-professional/cancer-statistics-for-the-uk

[2] Heaps JM, Fu YS, Montz FJ, Hacker NF, Berek JS. Surgical-pathologic variables predictive of local recurrence in squamous cell carcinoma of the vulva. Gynecol Oncol. 1990;38(3):309–14. 2227541 10.1016/0090-8258(90)90064-r

[3] Te Grootenhuis NC, Pouwer AW, de Bock GH, Hollema H, Bulten J, van der Zee AGJ, et al Prognostic factors for local recurrence of squamous cell carcinoma of the vulva: A systematic review. Gynecol Oncol. 2018;148(3):622–31. 10.1016/j.ygyno.2017.11.006 29137809

[4] Yap J, O’Neill D, Nagenthiran S, Dawson CW, Luesley DM. Current insights into the aetiology, pathobiology, and management of local disease recurrence in squamous cell carcinoma of the vulva. BJOG. 2017;124(6):946–54. 10.1111/1471-0528.14560 28081287

[5] Yap JK, Fox R, Leonard S, Ganesan R, Kehoe ST, Dawson CW, et al Adjacent Lichen Sclerosis predicts local recurrence and second field tumour in women with vulvar squamous cell carcinoma. Gynecol Oncol. 2016;142(3):420–6. 10.1016/j.ygyno.2016.06.019 27396942

[6] Vishnoi K, Mahata S, Tyagi A, Pandey A, Verma G, Jadli M, et al Cross-talk between Human Papillomavirus Oncoproteins and Hedgehog Signaling Synergistically Promotes Stemness in Cervical Cancer Cells. Sci Rep. 2016; 6:34377 10.1038/srep34377 27678330 PMC5039669

[7] Wang Y, Jin G, Li Q, Wang Z, Hu W, Li P, et al Hedgehog Signaling Non-Canonical Activated by Pro-Inflammatory Cytokines in Pancreatic Ductal Adenocarcinoma. J Cancer. 2016; 7(14):2067–76. 10.7150/jca.15786 27877222 PMC5118670

[8] Port RJ, Pinheiro-Maia S, Hu C, Arrand JR, Wei W, Young LS, et al Epstein-Barr virus induction of the Hedgehog signalling pathway imposes a stem cell phenotype on human epithelial cells. J Pathol. 2013; 231(3):367–77. 10.1002/path.4245 23934731

[9] Pasca di Magliano M, Hebrok M. Hedgehog signalling in cancer formation and maintenance. Nat Rev Cancer. 2003;3(12):903–11. 10.1038/nrc1229 14737121

[10] Xuan YH, Jung HS, Choi YL, Shin YK, Kim HJ, Kim KH, et al Enhanced expression of hedgehog signaling molecules in squamous cell carcinoma of uterine cervix and its precursor lesions. Mod Pathol. 2006;19(8):1139–47. 10.1038/modpathol.3800600 16778829

[11] Giroux Leprieur E, Vieira T, Antoine M, Rozensztajn N, Rabbe N, Ruppert AM, et al Sonic Hedgehog Pathway Activation Is Associated With Resistance to Platinum-Based Chemotherapy in Advanced Non-Small-Cell Lung Carcinoma. Clin Lung Cancer. 2016;17(4):301–8. 10.1016/j.cllc.2015.12.007 26762562

[12] Steg AD, Katre AA, Bevis KS, Ziebarth A, Dobbin ZC, Shah MM, et al Smoothened antagonists reverse taxane resistance in ovarian cancer. Mol Cancer Ther. 2012;11(7):1587–97. 10.1158/1535-7163.MCT-11-1058 22553355 PMC3392529

[13] Leonard S, Pereira M, Fox R, Gordon N, Yap J, Kehoe S, et al Over-expression of DNMT3A predicts the risk of recurrent vulvar squamous cell carcinomas. Gynecol Oncol. 2016;143(2):414–20. 10.1016/j.ygyno.2016.09.001 27623253

[14] Hirsch FR, Varella-Garcia M, Bunn PA Jr., Di Maria MV, Veve R, Bremmes RM, et al Epidermal growth factor receptor in non-small-cell lung carcinomas: correlation between gene copy number and protein expression and impact on prognosis. J Clin Oncol. 2003;21(20):3798–807. 10.1200/JCO.2003.11.069 12953099

[15] Collins SI, Constandinou-Williams C, Wen K, Young LS, Roberts S, Murray PG, et al Disruption of the E2 gene is a common and early event in the natural history of cervical human papillomavirus infection: a longitudinal cohort study. Cancer Res. 2009;69(9):3828–32. 10.1158/0008-5472.CAN-08-3099 19401452

[16] Fine JP, G. R. A proportional hazards model for the subdistribution of a competing risk. J Am Stat Assoc, 1999; 94:496–509.

[17] Gailani MR, Stahle-Backdahl M, Leffell DJ, Glynn M, Zaphiropoulos PG, Pressman C, et al The role of the human homologue of Drosophila patched in sporadic basal cell carcinomas. Nat Genet. 1996;14(1):78–81. 10.1038/ng0996-78 8782823

[18] Wang YF, Chang CJ, Lin CP, Chang SY, Chu PY, Tai SK, et al Expression of hedgehog signaling molecules as a prognostic indicator of oral squamous cell carcinoma. Head Neck. 2012;34(11):1556–61. 10.1002/hed.21958 22287313

[19] Hanna A, Shevde LA. Hedgehog signaling: modulation of cancer properies and tumor mircroenvironment. Mol Cancer. 2016;15:24 10.1186/s12943-016-0509-3 26988232 PMC4797362

[20] Powell JJ, Wojnarowska F. Lichen sclerosus. Lancet 1999;353(9166):1777–83. 10348006 10.1016/s0140-6736(98)08228-2

[21] Atwood SX, Sarin KY, Whitson RJ, Li JR, Kim G, Rezaee M, et al Smoothened variants explain the majority of drug resistance in basal cell carcinoma. Cancer cell 2015; 27(3): 342–353. 10.1016/j.ccell.2015.02.002 25759020 PMC4357167

[22] Atwood S X, Whitson RJ & Oro AE. Advanced treatment for basal cell carcinomas. Cold Spring Harb Perspect Med. 2014; 4(7):a013581 10.1101/cshperspect.a013581 24985127 PMC4066644

[23] Sharpe HJ, Pau G, Dijkgraaf GJ, Basset-Seguin N, Modrusan Z, Januario T, et al Genomic analysis of smoothened inhibitor resistance in basal cell carcinoma. Cancer Cell. 2015 3 9; 27(3):327–41. 10.1016/j.ccell.2015.02.001 25759019 PMC5675004

[24] Whitson RJ, Lee A, Urman NM, Mirza A, Yao CY, Brown AS, et al Noncanonical hedgehog pathway activation through SRF-MKL1 promotes drug resistance in basal cell carcinomas. Nat Med. 2018; 24(3):271–281. 10.1038/nm.4476 29400712 PMC5839965

[25] Samarzija I, Beard P. Hedgehog pathway regulators influence cervical cancer cell proliferation, survival and migration. Biochem Biophys Res Commun. 2012; 17;425(1):64–9. 10.1016/j.bbrc.2012.07.051 22820185

[26] Chen H, Wang J, Yang H, Chen D, Li P. Association between FOXM1 and hedgehog signaling pathway in human cervical carcinoma by tissue microarray analysis. Oncol Lett. 2016;12(4):2664–2673. 10.3892/ol.2016.4932 27698840 PMC5038455

[27] Sekulic A, Migden MR, Oro AE, Dirix L, Lewis KD, Hainsworth JD, et al Efficacy and safety of vismodegib in advanced basal-cell carcinoma. N Engl J Med. 2012;366(23):2171–9. 10.1056/NEJMoa1113713 22670903 PMC5278761

