# Supplementary figures and images for: Under expression of the Sonic Hedgehog receptor, Patched1 (PTCH1), is associated with an increased risk of local recurrence in squamous cell carcinoma of the vulva arising on a background of Lichen Sclerosus
Source: PLoS One. 2018 Oct 31;13(10):e0206553. doi: 10.1371/journal.pone.0206553 (PMC6209322; doi:10.1371/journal.pone.0206553)

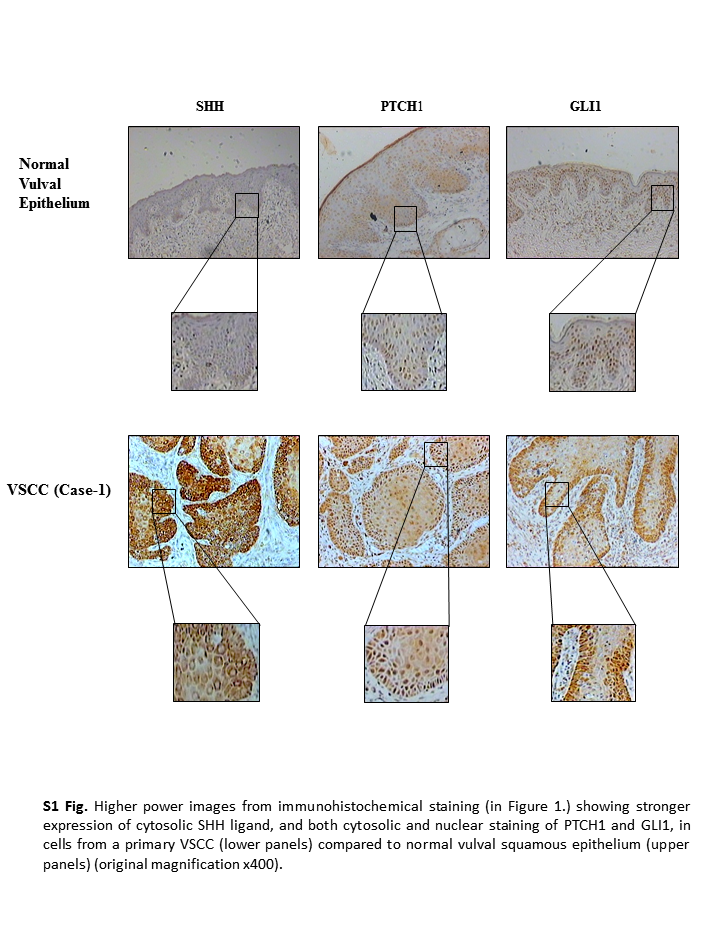

Supplement: S1 Fig — (TIF) [file pone.0206553.s001.tif]

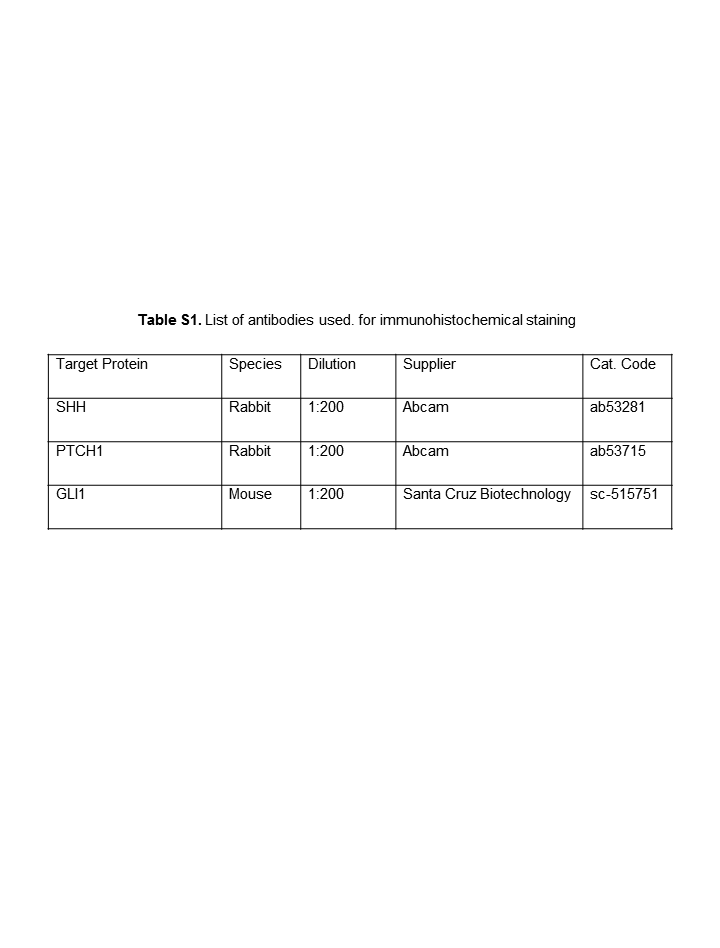

Supplement: S1 Table — (TIF) [file pone.0206553.s002.tif]

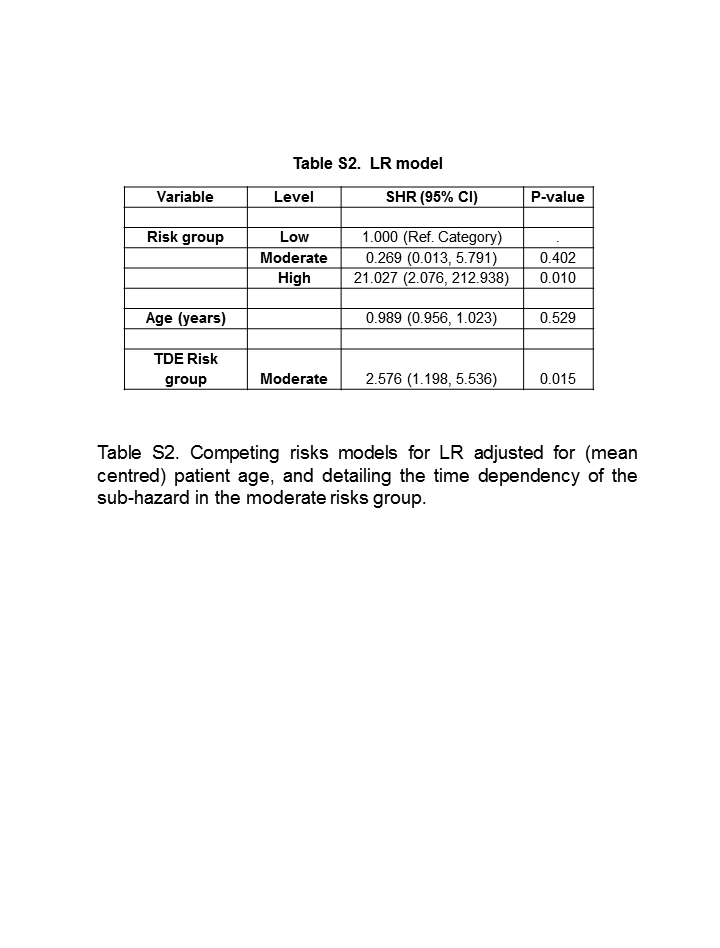

Supplement: S2 Table — (TIF) [file pone.0206553.s003.tif]
